# Pre-Randomization Predictors of Study Discontinuation in a Preclinical Alzheimer's Disease Randomized Controlled Trial

**DOI:** 10.14283/jpad.2024.136

**Published:** 2024-07-24

**Authors:** Rema Raman, K. Hussen, M.C. Donohue, K. Ernstrom, K.C. Holdridge, O. Langford, D.P. Molina-Henry, A.L. Pierce, J.R. Sims, A. Smith, R. Yaari, P.S. Aisen, R. Sperling, J.D. Grill, A4 Study Team

**Affiliations:** 1 Alzheimer's Therapeutic Research Institute, Keck School of Medicine, University of Southern California, San Diego, USA; 2 Eli Lilly and Company, Indianapolis, IN, USA; 3 Layton Aging and Alzheimer's Disease Center, Department of Neurology, Oregon Health and Science University, Portland, USA; 4 USF Health Byrd Alzheimer's Institute, Department of Psychiatry and Behavioral Neurosciences, University of South Florida, Tampa, USA; 5 Center for Alzheimer Research and Treatment, Department of Neurology, Brigham and Women's Hospital, Massachusetts General Hospital, Harvard Medical School, Boston, MA, USA; 6 Institute for Memory Impairments and Neurological Disorders, University of California Irvine, Irvine, USA; 7 Department of Psychiatry and Human Behavior, University of California Irvine, Irvine, USA; 8 Department of Neurobiology and Behavior, University of California Irvine, Irvine, USA

**Keywords:** Alzheimer's disease, preclinical AD, A4 study, study discontinuation, attrition, clinical trial

## Abstract

**Background:**

Participant discontinuation from study treatment in a clinical trial can leave a trial underpowered, produce bias in statistical analysis, and limit interpretability of study results. Retaining participants in clinical trials for the full study duration is therefore as important as participant recruitment.

**Objective:**

This analysis aims to identify associations of pre-randomization characteristics of participants with premature discontinuation during the blinded phase of the Anti-Amyloid treatment in Asymptomatic AD (A4) Study.

**Design:**

All A4 trial randomized participants were classified as having prematurely discontinued study during the blinded period of the study for any reason (dropouts) or completed the blinded phase of the study on treatment (completers).

**Setting:**

The trial was conducted across 67 study sites in the United States, Canada, Japan and Australia through the global COVID-19 pandemic.

**Participants:**

The sample consisted of all 1169 A4 trial randomized participants.

**Measurements:**

Pre-randomization demographic, clinical, amyloid PET and genetic predictors of study discontinuation were evaluated using a univariate generalized linear mixed model (GLMM), with discontinuation status as the binary outcome, each predictor as a fixed effect, and site as a random effect to account for differences among study sites in the trial. Characteristics significant at p<0.10 were then included in a multivariable GLMM.

**Results:**

Among randomized participants, 339 (29%) discontinued the study during the blinded period (median follow-up time in trial: 759 days). From the multivariable analysis, the two main predictors of study discontinuation were screening State-Trait Anxiety Inventory (STAI) scores (OR = 1.07 [95%CI = 1.02; 1.12]; p=0.002) and age (OR = 1.06 [95%CI = 1.03; 1.09]; p<0.001). Participants with a family history of dementia (OR = 0.75 [95%CI = 0.55; 1.01]; p=0.063) and APOE ε4 carriers (OR = 0.79 [95%CI = 0.6; 1.04]; p=0.094) were less likely to discontinue from the study, with the association being marginally significant. In these analyses, sex, race and ethnicity, cognitive scores and amyloid/tau PET scores were not associated with study dropout.

**Conclusions:**

In the A4 trial, older participants and those with higher levels of anxiety at baseline as measured by the STAI were more likely to discontinue while those who had a family history of dementia or were APOE ε4 carriers were less likely to drop out. These findings have direct implications for future preclinical trial design and selection processes to identify those individuals at greatest risk of dropout and provide information to the study team to develop effective selection and retention strategies in AD prevention studies.

## Introduction

It is increasingly recognized that challenging recruitment to clinical trials is a barrier to advances in treating Alzheimer's disease (AD) and AD-Related Dementias (ADRD) (1, 2, 3, 4, 5). Yet, while recent efforts by sponsors, academic leaders, and funding agencies recognize the need for improved methods of clinical research recruitment, particularly in the area of recruiting representative populations (6, 7) less attention has been paid to retaining participants once recruited to these trials. Challenges in retention can produce bias or error in scientific results and greater than expected dropout can leave a trial underpowered and even unethical (8, 9, 10). Retaining participants in clinical trials is therefore as important as recruitment. As with recruitment, scientific study is necessary to better elucidate the rates, risk factors, and impact of dropout in clinical trials (11, 12, 13).

Preclinical AD trials are a relatively new approach to testing AD therapies at a disease stage that may be most likely to benefit from disease-modifying treatments (14) and may be most likely to impact public health (15). Here, we examined the frequency and pre-randomization predictors of study discontinuation in one of the first and largest sporadic preclinical AD trials to date, the Anti-Amyloid treatment in Asymptomatic AD (A4) Study (16).

## Methods

The A4 Study (ClinicalTrials.gov identifier: NCT02008357) was a 240-week, randomized, placebo-controlled, double-blind, Phase III trial of solanezumab in older individuals who were not cognitively impaired at baseline but had elevated brain amyloid levels on screening positron-emission tomography (PET). This Study was approved by each study site's institutional review board. The design, methods and primary results have been published previously (17, 18).

### Study Participants, Design and Procedures

Intention-to-treat sample included 1169 randomized participants across 67 study sites in Australia, Canada, Japan, and the United States. Participants self-identified and self-referred for participation in the study. All participants and their study partners provided written informed consent prior to data collection, or any research activities being performed. Study dose was administered intravenously every 4 weeks. The double-blind phase of the study was 240 weeks with an open-label extension phase extending to 312 weeks.

### Measures collected at screening or baseline visits

The demographic, clinical, genetic, and biomarker variables used in this study were collected across up to six screening visits, conducted over a maximum of 12 weeks (90 days). A variety of demographic characteristics were collected at the initial screening visit through self-report. These included: participant's age at consent, sex, self-reported race, self-reported ethnicity, education (years), study partner type, marital status, and family history of dementia. An initial blood draw was used to perform genotyping for APOE ε4 carrier status (carrier/non-carrier) which was not disclosed to participants.

Validated instruments were used to assess participant-reported subjective cognitive complaints (Cognitive Function Index – Participant [CFI-PT]) (19) as well as study partner reporting of subjective cognitive performance (CFI – Study Partner [CFI-SP]) (20), depressive symptoms (Geriatric Depression Scale [GDS] (21)), and anxiety (State-Trait Anxiety Inventory [STAI] (22)). Objective cognitive performance was assessed using the Preclinical Alzheimer Cognitive Composite (PACC) (23). Only individuals with elevated brain amyloid, assessed through PET imaging, who met all other inclusion and exclusion criteria were eligible for randomization. We included baseline PET SUVr values as a measure in our analyses. Participants were informed of their amyloid PET biomarker eligibility through a structured disclosure process (24). Within 72-hours of learning their biomarker eligibility, participants underwent a telephone safety follow-up which included the Impact of Event Scale (IES), a measure of intrusive thoughts, that was included in our analyses.

Due to the sparseness of data, three variables were recoded. A participant's self-reported race and ethnicity were combined to create a race and ethnicity underrepresented group variable (RE-URG). Individuals who self-reported as being of Hispanic ethnicity or being one of the following races (American Indian or Alaska Native, Asian, Black or African American, more than one race) were classified as belonging to a RE-URG. Study partner relationship was classified into 3 mutually exclusive groups: spouse, adult child/child-in-law, other (friend, companion, other, other relative).

### Classification of Dropout status

Participants who prematurely discontinued from study treatment were required to immediately discontinue from the study. A randomized participant was considered a “dropout” in these analyses if they prematurely discontinued from treatment or halted participation for any other reason during the double-blind phase. If the individual completed the double-blind phase of the study on treatment, the participant was considered a “completer”.

### Statistical Analysis

These analyses included all randomized participants followed during the double-blind phase of the study. Characteristics of individuals who dropped out prematurely and those who remained in the study were described using frequencies with percentages for categorical variables and means with standard deviations for continuous variables.

A generalized linear model (GLM) without site as a random effect or a generalized linear mixed model (GLMM) with site as a random effect (if the site effect is observed to be statistically significant) was applied to assess the associations of each individual characteristic on study discontinuation status. Linearity assumption of continuous predictors in the model were checked by plotting the empirical logits (log odds of discontinuation within each quartile group) and the middle point of quartiles. Since this assumption was violated for education and baseline CFI study partner, these variables were recoded as follows: (1) Education was recoded as a categorical variable with 3 levels: High school graduate or less (12 years or less of formal education), some college (13–17 years of education), or professional degree (18 years or higher of formal education), (2) a median split was applied to categorize baseline CFI study partner score (i.e. below and equal to 1, and above 1), and (3) a tertile split was used for the baseline SUVr value.

Characteristics significant at the 0.10 level of significance in the univariate analysis were included in a multivariable model. If the assumption of linear odds was not met, the variables were transformed. Results are reported as odds ratios (OR) with corresponding 95% confidence intervals (CI). In the final multivariable model, only variables significant at the 0.10 level of significance were retained. All statistical analyses were conducted using the statistical software R version 4.2.2 (25).

## Results

Of the 1169 randomized participants, 339 (29%) prematurely dropped out of the blinded phase of the A4 trial. Of these, the two common reasons for dropout as reported by the study site are the participant being unwilling or unable to continue (56.3%) and adverse events (19.2%) (Table 1).

**Table 1:** Primary reason for study discontinuation (N=339)

Primary Reason	n (%)
Participant unwilling or unable to participate	191 (56.3%)
Adverse event	65 (19.2%)
Other	17 (5.0%)
Death	11 (3.2%)
COVID-19 pandemic disruption	10 (2.9%)
Safety risk	9 (2.7%)
Lost to follow up	9 (2.7%)
Perceived lack of efficacy	7 (2.1%)
Investigator recommendation	4 (1.2%)
Study partner unwilling or unable to participate	4 (1.2%)
Starting new trial	3 (0.9%)
Starting a new treatment for Alzheimer's disease	3 (0.9%)
Non-compliance	2 (0.6%)
Other non-site clinician recommendation	2 (0.6%)
Started prohibited medication	1 (0.3%)
Coordinating Center request	1 (0.3%)
Study terminated	0 (0.0%)

**Figure 1:**
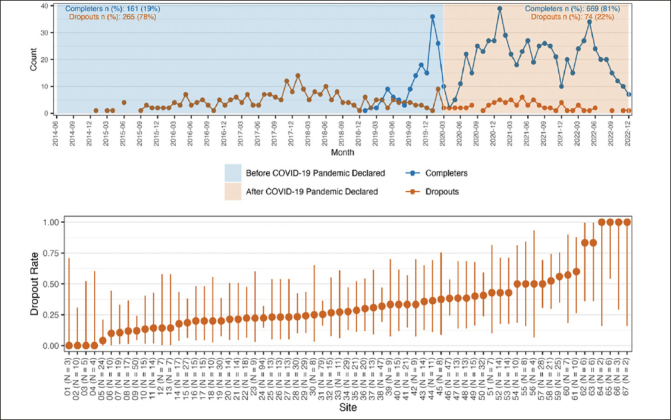
Counts and rates of premature study discontinuations during the blinded phase of the A4 trial Figure 1A (top row) describes the counts by study calendar time since the first randomization. The start of the global covid-19 pandemic is included as a vertical line on March 2023. Figure 1B (bottom row) describes the site-specific dropout rate sorted in ascending order. Sites are de-identified and included as sequential numbers from 1–67.

There was no evidence of an impact on dropout rates due to the interruption caused by the COVID-19 distancing measures. A flexible hiatus was permitted during the pandemic to try and limit attrition with a hiatus of up to 6–9 months in a few participants. Visual review show that dropout rates appear to be similar before and during the pandemic period of the study with consistent dropout numbers before and after the start of the pandemic through the end of the blinded phase of the study (Figure 1). There was, however, a statistically significant effect of site, based on a likelihood ratio test between the model with and without a random effect for this variable (LRT value=16.07, df=1, p<0.001). As a result, all subsequent analyses were conducted using the GLMM with a site-specific random effect.

**Figure 2:**
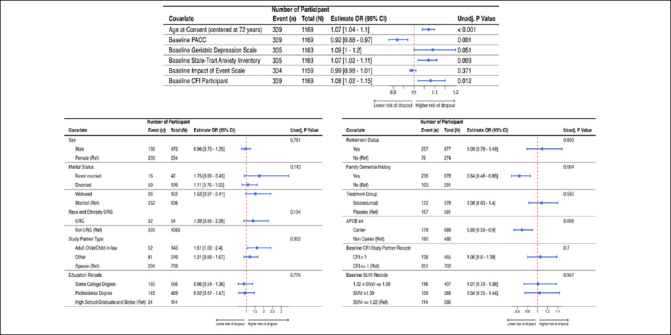
Results from the univariate generalized linear mixed model (GLMM) with discontinuation status as the outcome and site-specific random effect Plot includes the odds ratios with corresponding 95% confidence interval and the unadjusted p-value. Figure 2A (top row) provides the results for the continuous variables and Figure 2B (bottom row) provides the results for the categorical variables.

Table 2 shows the sample characteristics of randomized participants overall, and by discontinuation status. Figure 2 shows the results of the univariate analyses as forest plots with sample sizes, conditional odds ratios with 95% confidence intervals and unadjusted p-values. Family history of dementia, APOE ε4 carrier status, age (in years), baseline PACC, screening state trait anxiety inventory (STAI), baseline CFI participant and screening geriatric depression scale were all significantly associated with study discontinuation status at 0.1 level of significance.

**Table 2:** Pre-randomization characteristics of study sample by discontinuation status

Characteristics	Completers	Dropouts	Total
Demographic			
Age, mean (SD)	71.5 (4.5)	73.0 (5.4)	71.9 (4.8)
Female sex, n (%)	491 (59.2%)	203 (59.9%)	694 (59.4%)
Race			
American Indian/Alaska Native, n (%)	1 (0.1)	1 (0.3%)	2 (0.2%)
Asian, n (%)	18 (2.2%)	6 (1.8%)	24 (2.1%)
Black or African American, n (%)	14 (1.7%)	14 (4.1%)	28 (2.4%)
Native Hawaiian/Pacific Islander, n (%),	0 (0.0 %)	0 (0.0%)	0 (0.0%)
More than One Race, n (%)	6 (0.7%)	2 (0.6%)	8 (0.7%)
White, n (%)	786 (94.7%)	314 (92.6%)	1100 (94.1%)
Hispanic ethnicity, n (%)	24 (2.9%)	10 (2.9%)	34 (2.9%)
Race and Ethnic Underepresented Group (RE-URG)			
Yes, n (%)	62 (7.5%)	32 (9.6%)	94 (8.1%)
No, n (%)	760 (92.5%)	303 (90.4%)	1063 (91.9%)
Study partner type			
Spouse, n (%)	538 (65.8%)	201 (60.2%)	739 (64.1%)
Adult child, n (%)	91 (11.1%)	52 (15.6%)	143 (12.4%)
Other, n (%)	189 (23.1%)	81 (24.3%)	270 (23.4%)
Married, n (%)	604 (72.8%)	232 (68.4%)	836 (71.5%)
Family history AD, n (%)	642 (77.3%)	236 (69.6%)	878 (75.1%)
Randomized to Solanezumab	406 (48.9%)	172 (50.7%)	578 (49.4%)
Clinical			
CFI participant, mean (SD)	2.3 (2.1)	2.6 (2.3)	2.4 (2.2)
CFI study partner, mean (SD)	1.4 (2.0)	1.6 (2.0)	1.5 (2.0)
GDS, mean (SD)	1.0 (1.4)	1.2 (1.5)	1.1 (1.4)
STAI, mean (SD)	9.7 (3.0)	10.3 (3.2)	9.9 (3.1)
IES, mean (SD)	10.5 (10.9)	9.6 (10.4)	10.2 (10.8)
PACC, mean (SD)	0.1 (2.6)	−0.4 (2.7)	0.0 (2.7)
Genetic			
APOE ε4 Carrier, n (%)	510 (61.4%)	179 (52.8%)	689 (58.9%)
Biomarker			
PET SUVr, mean (SD)	1.3 (0.2)	1.3 (0.2)	1.3 (0.2)

**Figure 3:**
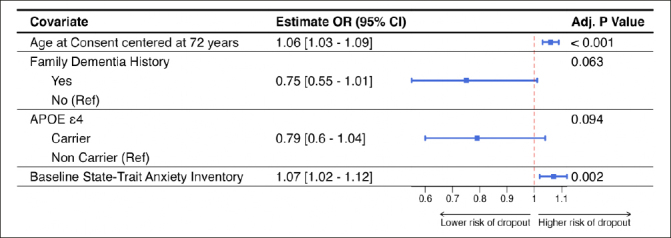
Results from the multivariable generalized linear mixed model with discontinuation status as the outcome and site-specific random effect Plot includes the conditional odds ratios with corresponding 95% confidence interval and the unadjusted p-value.

Figure 3 shows the results of the multivariable logistic regression model including only those variables that were significant at 0.10 level of significance. Older study participants (OR = 1.06 [95%CI = 1.03; 1.09]; p<0.001) and higher levels of screening state trait anxiety inventory (OR = 1.07 [95%CI = 1.02; 1.12]; p=0.002) were significantly associated with study dropout in this model. Having a known family history of dementia (OR = 0.75 [95%CI = 0.55; 1.01]; p=0.063) and being an APOE ε4 carrier (OR = 0.79 [95%CI = 0.6; 1.04]; p=0.094) were marginally associated with study dropout.

## Discussion

The 29% study discontinuation rate observed in the A4 study was less than the 30% attrition rate projected in the design and power calculations. Even with the burden of monthly infusion visits, a duration of 240 weeks and social distancing measures due to a global pandemic that paused the study for several months, the rate of study dropout was relatively low with an average of 10 participants per quarter. We did not observe an increase in dropout during the study pause that occurred during the global COVID-19 pandemic. This attrition rate of 6.4% per year over 4.5 years observed in this preclinical AD trial is lower than the 20% per year attrition rate observed in (short) MCI and AD dementia trials 26 and does not appear to be associated with trial duration as was observed in these trials (27).

We observed significant site differences in the rates of study discontinuation. Hence, the analysis model included site as a random effect, thereby considering each of the A4 study sites as a random sample of a population of hypothetical sites. Differences in study dropout by site type (commercial versus academic) has been observed in MCI studies (28). Ongoing analyses will evaluate whether study site characteristics are related to attrition in A4.

In this study, we found that increased age and higher scores at screening on the State-Trait Anxiety Inventory (STAI) score were statistically significant predictors of overall study dropout. For each year increase in age, there was a 6% increase in the odds of study dropout, while a unit increase in STAI score resulted in a 7% increase in the odds of study dropout. These findings are consistent with observations from meta-analyses conducted in AD dementia trials (29, 30). As for anxiety, it is important to note that the observed scores for STAI were infrequently considered clinically meaningful and changes in score were, in fact, no different for those eligible due to having elevated brain amyloid compared to those who were deemed ineligible for having not having elevated brain amyloid. Thus, the observed relationship was likely based on the large sample size and the opportunity to use these scores to identify those at risk of dropout may be of limited value.

The analyses also identified individuals who had a family history of Alzheimer's disease and individuals who were APOE ε4 carriers as having a decreased odds of trial dropout of 25% and 21% respectively; though these associations were only marginally significant. Increased genetic risk or family history may be motivating factors for participants to remain in the study especially in the preclinical stage of the disease. Interestingly, while participants self-reported their family history of dementia, the trial protocol did not reveal genetic testing results. While some participants may have known their APOE status at entry (e.g., through direct-to-consumer testing options), we unfortunately did not collect this information in the trial to assess this. Other baseline characteristics such as education, baseline cognitive and functional scores, study partner type, and depression scores did not reach statistical significance once included in the multivariable model, suggesting that these baseline factors did not contribute to participant dropout.

This analysis has one major limitation. The A4 study sample was not racially and ethnically diverse, with only 94 (8.1%) of the randomized participants self-identifying as belonging to a racial or ethnic underrepresented group (RE-URG). We observed a nominally higher dropout rate among participants from RE-URG (32/94; 34%) compared to those not from a RE-URG (303/1063; 28.5%). The small sample size limits opportunity to evaluate the meaningfulness of this difference and exploration of interactions between race and ethnicity and other factors, such as education.

Ongoing work aims to identify additional longitudinal predictors of premature study discontinuation as well as predictors of non-compliance to intervention to help inform the design and conduct of future AD prevention trials. This work also focuses on identifying site specific predictors of preclinical AD trial retention and evaluating whether predictors are similar across important subpopulations of interest. Factors of consideration include but will not be limited to the number randomized, site type (academic vs non-academic), Principal Investigator type (neurologist, psychiatrist, other), geography, local restrictions during COVID, and research staff turnover.

In conclusion, the results from the analyses of one of the first and largest completed preclinical AD trials identified two pre-randomization factors that predicted study dropout vs. completion. These findings have implications and provide important guidance to the design and conduct of future preclinical AD trials. Identifying baseline characteristics associated with dropout and completion may provide investigators the opportunity to establish recruitment and retention strategies that account for these factors and counter the foreseeable attrition thereby minimizing bias and maintaining overall study power.

*Funding:* The A4 study was supported by a public-private-philanthropic partnership which included funding from the National Institute of Aging of the National Institutes of Health (R01 AG063689, U19AG010483 and U24AG057437), Eli Lilly (also the supplier of active medication and placebo), the Alzheimer's Association (LEARN, NIRG-12-243012), the Accelerating Medicines Partnership through the Foundation for the National Institutes of Health, the GHR Foundation, the Davis Alzheimer Prevention Program, the Yugilbar Foundation, an anonymous foundation, and additional private donors to Brigham and Women's Hospital, with in-kind support from Avid Radiopharmaceuticals, Cogstate, Albert Einstein College of Medicine and the Foundation for Neurologic Diseases. Open access funding provided by SCELC, Statewide California Electronic Library Consortium.

*Conflicts of interest:* RR has received research support from the National Institutes of Health (NIH), the Alzheimer's Association, American Heart Association, Eli Lilly and Eisai. KCH, RY, and JRS are employees and minor shareholders of Eli Lilly and Company. KH, OL and KE have received research support from the National Institutes of Health (NIH), the Alzheimer's Association, American Heart Association, and Eisai. MD has received research funding from the National Institutes of Health, Janssen, Eli Lilly, and Eisai, reports consulting fees from Roche and his spouse is a full-time employee of Janssen. DMH is part of the AHEAD 3–45 Study team. AHEAD A3-45 Study is a public-private partnership with funding from the National Institutes of Aging/NIH, Eisai, and Co., GHR Foundation, Alzheimer's Association, and philanthropic donors. Additionally, she receives direct funding from the Alzheimer's Association and American Heart Association. ALP has received research support from the National Institutes of Health (NIH), Alzheimer's Association, Hart Family Foundation, Alector, Cognition Therapeutics, Eisai, Eli Lilly, and Vivoryon. She has served as a consultant for Medscape. AGS has received research support from the National Institutes of Health (NIH), the Alzheimer's Association, Eli Lilly, Biogen, Eisai, Janssen, Vivoryon, Cassava, Bristol Myers Squibb, and the American College of Radiology. PSA has received grants or contracts from the National Institutes of Health (NIH), Alzheimer's Association, Foundation for NIH (FNIH), Lilly, Janssen and Eisai and consulting fees from Merck, Biogen, AbbVie, Roche, and Immunobrain Checkpoint. RAS reports grant support from Eisai, and Eli Lilly and reported serving as a consultant for AbbVie, AC Immune, Alector, Bristol-Myers-Squibb, Ionis, Janssen, Genentech, Merck, Prothena, Roche, and Vaxxinity. JG has received research support from the National Institutes of Health (NIH), the Alzheimer's Association, BrightFocus Foundation, Eli Lilly, Biogen, Genentech, and Eisai. He has received personal fees for providing consulting to SiteRx and editorial service to Alzheimer's & Dementia.

*Ethical Standards:* Approval from an institutional review board or ethics committee was obtained at each of the sites. All participants and their study partners provided written informed consent prior to data collection, which included consent for data sharing.

## References

[1] Aisen PS, Jimenez-Maggiora GA, Rafii MS, Walter S, Raman R (2022). Early-stage alzheimer disease: Getting trial-ready. Nat Rev Neurol.

[2] Elliott CL (2020). Together we make the difference: National strategy for recruitment and participation in alzheimer's and related dementias clinical research. Ethn Dis.

[3] Fargo KN, Carrillo MC, Weiner MW, Potter WZ, Khachaturian Z (2016). The crisis in recruitment for clinical trials in alzheimer's and dementia: An action plan for solutions. Alzheimers Dement.

[4] Langbaum JB, Zissimopoulos J, Au R (2023). Recommendations to address key recruitment challenges of alzheimer's disease clinical trials. Alzheimers Dement.

[5] Watson JL, Ryan L, Silverberg N, Cahan V, Bernard MA (2014). Obstacles and opportunities in alzheimer's clinical trial recruitment. Health Aff (Millwood).

[6] Manly JJ, Gilmore-Bykovskyi A, Deters KD (2021). Inclusion of underrepresented groups in preclinical alzheimer disease trials-opportunities abound. JAMA Netw Open.

[7] Raman R, Aisen PS, Carillo MC (2022). Tackling a major deficiency of diversity in alzheimer's disease therapeutic trials: An CTAD task force report. J Prev Alzheimers Dis.

[8] Halpern SD, Karlawish JHT, Berlin JA (2002). The continuing unethical conduct of underpowered clinical trials. JAMA.

[9] Grill JD, Kwon J, Teylan MA (2019). Retention of alzheimer disease research participants. Alzheimer Dis Assoc Disord.

[10] National Research Council 2. The prevention and treatment of missing data in clinical trials. Washington, DC: The national academies press.

[11] Stites SD, Turner RS, Gill J (2021). Research attitudes questionnaire scores predict alzheimer's disease clinical trial dropout. Clin. Trials.

[12] Crane PK, Doody RS (2009). Donepezil treatment of patients with MCI: A 48-week randomized, placebo- controlled trial. Neurology.

[13] Grill JD, Raman R, Ernstrom K, Aisen P, Karlawish J (2013). Effect of study partner on the conduct of alzheimer disease clinical trials. Neurology.

[14] Sperling RA, Karlawish J, Johnson KA (2013). Preclinical alzheimer disease-the challenges ahead. Nat. Rev. Neurol.

[15] Brookmeyer R, Abdalla N, Kawas CH, Corrada MM (2018). Forecasting the prevalence of preclinical and clinical alzheimer's disease in the united states. Alzheimers Dement.

[16] Sperling RA, Rentz DM, Johnson KA (2014). The A4 study: Stopping AD before symptoms begin?. Sci. Transl. Med.

[17] Sperling RA, Donohue MC, Raman R (2020). Association of factors with elevated amyloid burden in clinically normal older individuals. JAMA Neurol.

[18] Sperling RA, Donohue MC, Raman R (2023). Trial of solanezumab in preclinical alzheimer's disease. N. Engl. J. Med.

[19] Walsh SP, Raman R, Jones KB, Aisen PS, Alzheimer's Disease Cooperative Study Group (2006). ADCS prevention instrument project: The mail-in cognitive function screening instrument (MCFSI). Alzheimer Dis. Assoc. Disord.

[20] Amariglio RE, Donohue MC, Marshall GA (2015). Tracking early decline in cognitive function in older individuals at risk for alzheimer disease dementia: The alzheimer's disease cooperative study cognitive function instrument. JAMA Neurol.

[21] Sheikh JI, Yesavage JA, Brooks JO (1991). Proposed factor structure of the geriatric depression scale. Int. Psychogeriatr.

[22] Marteau TM, Bekker H (1992). The development of a six-item short-form of the state scale of the spielberger state-trait anxiety inventory (STAI). Br. J. Clin. Psychol.

[23] Donohue MC, Sperling RA, Salmon DP (2014). The preclinical alzheimer cognitive composite: Measuring amyloid-related decline. JAMA Neurol.

[24] Grill JD, Raman R, Ernstrom K (2020). Short-term psychological outcomes of disclosing amyloid imaging results to research participants who do not have cognitive impairment. JAMA Neurol.

[25] R core team. (2023). R: A language and environment for statistical computing.

[27] Ritchie M, Gillen DL, Grill JD (2023). Estimating attrition in mild-to-moderate alzheimer's disease and mild cognitive impairment clinical trials. Alzheimers Res. Ther.

[28] Edland SD, Emond JA, Aisen PS, Petersen RC (2010). NIA-funded alzheimer centers are more efficient than commercial clinical recruitment sites for conducting secondary prevention trials of dementia. Alzheimer Dis. Assoc. Disord.

[29] Crimin Kea (2021). Identifying predictive factors of patient dropout in alzheimer's disease clinical trials. Alzheimer's and Dementia.

[30] Henley DB, Sundell KL, Sethuraman G, Schneider LS (2015). Adverse events and dropouts in alzheimer's disease studies: What can we learn?. Alzheimers Dement.

